# Implementing pain competencies in Canadian physiotherapy education: Challenges, barriers, and opportunities

**DOI:** 10.1080/24740527.2025.2574969

**Published:** 2025-12-15

**Authors:** Nathan Augeard, Christian Longtin, André Bussières, Geoff Bostick, Aliki Thomas, Jordan Miller, Yannick Tousignant-Laflamme, David Walton, Anne Hudon, Lynn Cooper, Lesley Singer, Fatima Amari, Timothy H. Wideman

**Affiliations:** a School of Physical and Occupational Therapy, Faculty of Medicine and Health Sciences, McGill University, Montreal, Quebec, Canada; b Département Chiropratique, Université du Québec à Trois-Rivières, Trois-Rivières, Quebec, Canada; c Department of Physical Therapy, University of Alberta, Edmonton, Alberta, Canada; d Centre de Recherche Interdisciplinaire en Réadaptation du Montréal Métropolitain, Montreal, Quebec, Canada; e Institute of Health Sciences Education, McGill University, Montreal, Quebec, Canada; f School of Rehabilitation Therapy and Health Services and Policy Research Institute, Queen’s University, Montreal, Quebec, Canada; g Programme de physiothérapie, Université de Sherbrooke, Sherbrooke, Quebec, Canada; h School of Physical Therapy, Western University, London, Ontario, Canada; i Programme de physiothérapie, École de Réadaptation, Faculté de Médecine, Université de Montréal, Montreal, Quebec, Canada; j Canadian Injured Workers Alliance, Toronto, Canada; k Quebec Pain Research Network, Chronic Pain Network, Montreal, Canada

**Keywords:** Pain management education, competency-based education, physiotherapy curriculum, implementation science, faculty development

## Abstract

**Background:**

The Pain Education in Physiotherapy (PEP) competency profile provides a structured framework for integrating pain management competencies into Canadian physiotherapy (PT) curricula. Despite widespread endorsement, the integration of pain management competencies into PT curricula remains inconsistent. Identifying the barriers and enablers to implementation is essential for developing strategies that support students in achieving these competencies.

**Objective:**

This study explored factors influencing the implementation of the PEP competency profile in Canadian PT programs and identified key challenges and opportunities for improving integration.

**Methods:**

A qualitative description study was conducted using five focus groups with 23 participants, including pain educators and program directors from 13 entry-level PT programs in Canada. Data were analyzed using the Consolidated Framework for Implementation Research to identify multilevel barriers and facilitators.

**Results:**

Participants recognized the value of the PEP competency profile in enhancing pain education but highlighted three key challenges: (1) a lack of structured guidance for teaching and assessment, (2) an overreliance on faculty champions rather than systemic institutional support, and (3) the absence of rigorous assessment approaches. Participants expressed uncertainty about integrating competencies within existing curricula, emphasizing the need for national collaboration, faculty development, and shared resources. The iterative, decentralized nature of curriculum change further complicated efforts to achieve consistent integration.

**Conclusion:**

Sustainable implementation of the PEP competencies requires structured guidance, institutional commitment, and adapted assessment strategies. Addressing these barriers through national-level collaboration, accreditation alignment, and faculty support is critical to ensure that PT graduates develop the necessary competencies for high-quality pain management.

## ABSTRAIT

**Contexte:** Le profil de compétences Pain Education in Physiotherapy (PEP) offre un cadre structuré pour l’intégration des compétences en gestion de la douleur dans les programmes canadiens de physiothérapie (PT). Malgré un large endossement, l’intégration de ces compétences demeure inégale. L’identification des obstacles et des facilitateurs à l’implantation est essentielle pour élaborer des stratégies soutenant les étudiants dans l’acquisition de ces compétences.

**Objectifs:** Cette étude visait à explorer les facteurs influençant l’implantation du profil de compétences PEP dans les programmes canadiens de PT et à identifier les principaux défis et opportunités pour améliorer son intégration.

**Méthodes:** Une étude descriptive qualitative a été réalisée à partir de cinq groupes de discussion réunissant 23 participants, incluant des éducateurs en douleur et des directeurs de programme provenant de 13 programmes de PT au Canada. Les données ont été analysées à l’aide du Consolidated Framework for Implementation Research (CFIR) afin d’identifier les obstacles et les facilitateurs à plusieurs niveaux.

**Résultats:** Les participants ont reconnu la valeur du profil de compétences PEP pour renforcer l’éducation à la douleur, mais ont souligné trois défis principaux : (1) l’absence de lignes directrices structurées pour l’enseignement et l’évaluation, (2) une dépendance excessive envers certains enseignants mobilisés plutôt qu’un soutien institutionnel systémique, et (3) le manque d’approches d’évaluation rigoureuses. Les participants ont exprimé des incertitudes quant à l’intégration des compétences dans les curriculums existants, insistant sur la nécessité d’une collaboration nationale, du développement du corps professoral et du partage de ressources. Le caractère itératif et décentralisé du changement curriculaire a également compliqué les efforts visant une intégration cohérente.

**Conclusion:** Une mise en œuvre durable des compétences PEP exige des lignes directrices structurées, un engagement institutionnel et des stratégies d’évaluation adaptées. La levée de ces obstacles par une collaboration nationale, une harmonisation avec les exigences d’agrément et un soutien au corps professoral est essentielle pour garantir que les diplômés en PT développent les compétences nécessaires à une gestion de la douleur de haute qualité.

## Introduction

Pain remains a leading cause of disability worldwide, contributing significantly to societal and economic burdens.^1–6^ Despite the critical need for effective pain management,^1,2^ many patients report suboptimal care stemming from health care providers’ limited competencies in areas such as empathy, communication, and validation.^7–12^ These gaps often result in stigma, misunderstanding, and missed opportunities for comprehensive pain management.^7–12^ Physiotherapists (PTs) are well positioned to address these issues as important members of interprofessional care teams for people living with pain, through their nonpharmacological approach, use of movement-based strategies, focus on self-management, and role in patient education.^13–15^ Ensuring that PTs are adequately prepared to manage pain upon entering clinical practice is essential to improving patient outcomes and mitigating the broader impact of pain-related suffering and disability.^9,16–18^

Entry-level education plays a pivotal role in preparing PTs with the foundational competencies required for effective pain management.^19–25^ It is during this formative stage that future clinicians begin to develop the conceptual frameworks, clinical reasoning habits, and interpersonal approaches that shape long-term practice.^19–25^ However, important inconsistencies remain across PT programs in how pain management is taught and assessed.^26–34^ Curriculum variations, particularly in the integration of interpersonal skills and critical competencies, raise concerns about whether graduates are sufficiently and consistently prepared to address the complexities of pain.^26–34^ These disparities mirror broader challenges in pain education across health professions and highlight the need for a standardized, comprehensive approach to pain management education.^21,25–33,35–47^

In Canada, PT education is delivered through 15 accredited university programs offering a 2-year, course-based master’s degree that usually follows completion of an undergraduate program in a related field. The Pain Education in Physiotherapy (PEP) competency profile was developed to establish a national standard by providing a shared goal for pain management education in this context.^48^ The PEP profile defines essential competencies required for entry-level practice, ranging from technical skills such as conducting comprehensive pain assessments to interpersonal abilities like supporting patient autonomy, with a focus on complex pain, understood as involving persistent, multidimensional drivers, though the competencies also apply across acute and chronic settings.^48^ It was developed through a national consensus process involving educators, clinicians, individuals with lived experience, and PT students, including a stakeholder workshop and a two-round Delphi with 14 of the 15 Canadian programs.^48^ Though the profile has gained national consensus as a guiding framework for pain education, implementing it into diverse educational contexts remains a challenge.^49,50^ In this study, implementation refers to integrating these competencies into program and course learning outcomes, developing teaching and assessment strategies to support student competency development, and embedding them within broader curricular structures. Although some prior work has described efforts to embed pain education in PT curricula,^51^ few studies have examined how endorsed competencies are operationalized or what institutional and pedagogical factors shape their implementation.

This study explores the barriers and facilitators to implementing the PEP competency profile into Canadian entry-level PT programs. By identifying institutional, contextual, and individual factors that affect implementation, this research seeks to provide actionable insights for educators, program directors, and policymakers in university settings. These findings are critical to advancing pain management education, fostering consistency across PT programs, and ensuring that graduates are well prepared to deliver high-quality, patient-centered care for individuals living with pain. By examining the implementation of a nationally endorsed competency profile, this study addresses a key gap in the literature and provides insight into the conditions necessary for scalable, sustainable curricular change.

## Methods

### Creation of a steering group

A steering group of nine members, including people living with pain (L.C., L.S.), pain educators (D.W., J.M., T.W., Y.T.L.), an expert in qualitative research (G.B.), and implementation science experts (A.B., A.T.), oversaw all aspects of the study. All members of the steering group had equal involvement in the planning and decision making related to this project.

### Framework

The Consolidated Framework for Implementation Research (CFIR) was used to explore the barriers and facilitators to implementing the PEP competency profile into entry-level PT programs in Canada.^52–56^ This framework is widely used in implementation science to assess factors influencing the adoption of evidence-based practices.^52,53^ It comprises five domains: innovation characteristics, outer setting, inner setting, characteristics of individuals, and implementation process.^52,53^ In this study, these domains were operationalized as follows: *innovation* referred to the PEP competency profile. The *outer setting* was intended to capture influences outside of the PT program, such as university-level factors, accreditation standards, or societal pressures. The *inner setting* referred to factors within the PT program, at the level of directors and educators. The *individuals* domain encompassed the distinct roles involved with education, including but not limited to educators, curriculum designers, program directors, students, and teaching assistants. The *implementation process* domain covered the steps and process involved in the implementation of the PEP competencies within the local PT curriculum. Finally, the CFIR *outcomes* addendum included anticipated outcomes (adoptability, implementability, sustainability) and actual outcomes (adoption, implementation, sustainment) of such implementation efforts.^56^ Given the participatory approach used in developing the PEP competency profile, many individuals involved in its creation had already begun informal implementation. This addition allowed the study to capture early perspectives on implementation progress alongside barriers and facilitators, providing a more dynamic picture of change.

Though the CFIR is widely used in health care implementation research, its application in health professions education (HPE) remains limited. Scholars in HPE are increasingly adopting implementation frameworks to examine challenges in curriculum change and competency implementation.^57^ This study provided an opportunity to apply the CFIR to a broader, multifaceted process: the implementation of competencies into PT curricula. Unlike discrete interventions, such as implementing a specific treatment, curriculum change involves multiple stakeholders, institutional structures, and varying levels of adoption across programs. Employing the CFIR in this context can offer insight into whether its constructs adequately capture the complexity of curriculum implementation or require adaptation for competency-based education.

### Study design

This study employed a qualitative descriptive design, an approach well suited to applied research aimed to capture participants’ perceptions and experiences and the contextual factors influencing implementation, with minimal inference.^58–61^ This methodology prioritizes a clear, low-inference account of participants’ views, grounded in their own language and context. Though the study acknowledges that participants’ accounts are shaped by institutional and social contexts, consistent with the recognition that knowledge is situated,^62^ the analytic approach remained descriptive, focused on producing a practical and contextually grounded account of perceived barriers and facilitators to implementation.^62^ To ensure methodological rigor and transparency, we adhered to the Consolidated Criteria for Reporting Qualitative Research (checklist in Supplementary Material 1).^63^

### Study participants

Participants included pain educators, program directors, and others involved in pain management education within entry-level PT programs across Canada, whose roles in curriculum development, instructional delivery, and institutional decision making provided essential insights into the barriers and facilitators to implementing the PEP competency profile. Recruitment was conducted via e-mail invitations sent to individuals from all 15 accredited Canadian PT programs, identified through past collaborations, professional networks, and institutional websites. A snowball sampling strategy was used, allowing initial participants to refer others with relevant expertise (e.g., inviting the participation of other pain educators from their institution).^64^ Recruitment continued until data saturation was reached, meaning no new themes emerged from additional focus groups (FGs), ensuring both conceptual breadth and interpretive depth.^65^ The number and composition of FGs were determined pragmatically, based on who responded, their linguistic preferences, and their availability to attend the discussion sessions.

### Data collection

Five FGs were conducted to explore diverse perspectives on the barriers and facilitators to implementing the PEP competency profile.^66–69^ This method fostered rich, interactive dialogue, allowing participants to build on each other’s ideas and collectively reflect on shared and unique challenges.^66–69^ The collaborative nature of FGs was particularly well suited to examining the interplay of institutional, individual, and contextual factors influencing implementation.^66–69^ A discussion guide was developed by the steering group to align with study objectives and CFIR constructs, ensuring a comprehensive examination of implementation factors. The guide was refined iteratively through the sessions, enhancing clarity, relevance, and methodological rigor.^66–69^ Each FG included two discussion sessions, lasting less than 2 h and held a maximum 2 weeks apart, minimizing participant burden while allowing for reflection between sessions. The first session explored the participants’ existing knowledge, perceptions, and current practices related to pain management education and the PEP competency profile, and the second session shifted toward envisioning and analyzing the practicalities, resources, institutional dynamics, and external influences involved in implementing the profile. Discussions were conducted virtually via Zoom for accessibility and participants were grouped by role, with separate sessions for educators and program directors to encourage open dialogue and reduce power dynamics. Sessions were held in English and French to accommodate language preferences. All FGs were conducted by the lead author (N.A.), a PT and PhD candidate in rehabilitation sciences with prior experience in qualitative research and pain education. At the time of data collection, the lead author was affiliated with McGill University and actively involved in national pain education initiatives. The lead author had previously collaborated or interacted with approximately a third of the participants through professional networks, research collaborations, or educational initiatives. These prior relationships were collegial rather than supervisory, and participants were aware of the lead author’s and the steering group’s research backgrounds and interests in pain management education. To mitigate potential influence on participant responses, N.A. emphasized the voluntary nature of participation and the confidentiality of the sessions. The shared professional background and familiarity with the topic were perceived as strengths in building rapport and facilitating open dialogue. Discussions were recorded, transcribed verbatim, and supplemented with field notes to ensure accuracy and capture nuances in participants’ contributions.

### Data analysis

The data analysis was guided by the CFIR, which provided a structured framework to systematically organize and interpret findings.^52,53^ The analysis followed a multistep process beginning with deductive coding, applying the CFIR’s five domains, 39 constructs, and 13 outcomes to the transcripts.^52,53^ This was followed by inductive coding of the data within each CFIR domain to identify themes.^52,53^ To ensure rigor and trustworthiness, two independent coders (C.L., N.A.) analyzed transcripts, meeting regularly to resolve discrepancies and refine the coding framework. Disagreements were resolved through discussion. Codes were iteratively refined to capture the complexity of participants’ experiences and implementation factors. NVivo 15 software facilitated data organization by tracking the evolution of codes and themes and ensuring consistency in analysis.^70^

Three steering group members (A.B., G.B., T.W.) provided ongoing feedback to ensure alignment with study objectives and enhance credibility. Reflexivity was maintained throughout, with the research team examining how their professional roles, assumptions, and prior relationships with participants may have shaped data collection and interpretation. This process fostered ongoing reflexivity during coding and theme development, supporting awareness of how the researchers’ positionalities, assumptions, and interpretive lenses may have shaped the analysis and helped prevent the uncritical privileging of particular viewpoints or imposition of preexisting frames. Reliability was reinforced through iterative review cycles, revisiting coded data as patterns emerged to ensure a comprehensive and accurate representation of participants’ perspectives. Themes were developed and embedded within the overarching CFIR domains, ensuring that findings remained grounded in the framework while allowing for novel insights into the factors shaping the adoption and implementation of the PEP competency profile.

### Ethical considerations

Ethics approval for this study was obtained from McGill University’s Institutional Review Board (Approval No. A11-E78-19A). Written informed consent was obtained from all participants prior to the FGs. To protect confidentiality, all identifying information was removed during transcription, and pseudonyms were assigned to anonymize responses.

## Results

### Participants

A total of 23 participants out of the 36 invited participated in this study (64%), including 15 educators and eight PT program directors. Overall, 13 of the 15 PT programs in Canada were represented (87%). The sample included participants from six different provinces, reflecting a broad cross section of Canadian PT education and capturing perspectives from both anglophone and francophone programs. Participants were grouped into five FGs (between 3 and 5 participants per group), each scheduled for two sessions. Three groups were composed of educators and two included program directors. In total, three FGs were conducted in English and two in French. Attendance varied, with 12 participants attending both sessions and 11 participants attending only one of the two sessions. No participants withdrew from the study after consenting to participate. Some invitees were unable to attend due to scheduling conflicts or limited availability.

### Overview of findings

**Figure 1.**
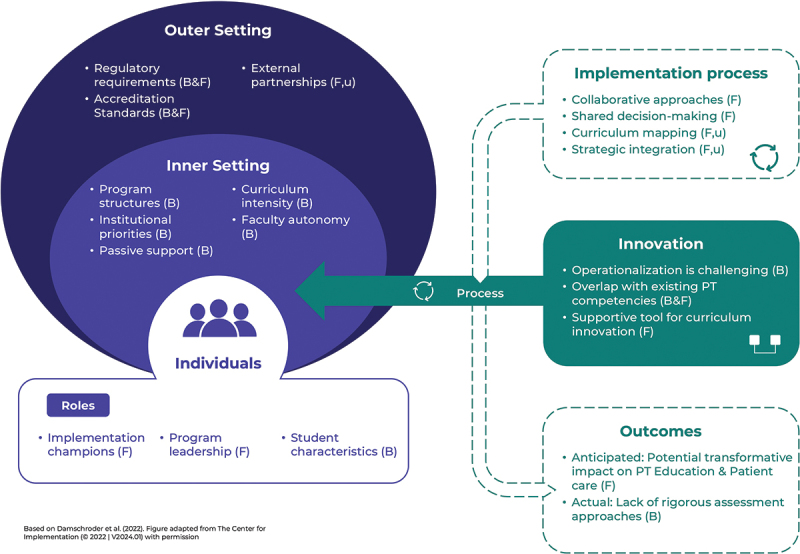
Summary of barriers and facilitators to implementation based on the CFIR domains and outcomes. F = facilitator, B = barrier, u = underutilized.

This study identified six overarching themes and 14 subthemes, spanning five CFIR domains, 34 CFIR constructs, and six outcome categories, illustrating the institutional, individual, and contextual factors influencing the implementation of the PEP competency profile in PT programs (Figure 1). A detailed breakdown of the results is provided in Supplementary Material 2 to offer additional context and transparency regarding the analytical process.

### Innovation domain

The first theme highlights that the PEP competency profile was viewed by participants as a flexible and structured tool that could support curriculum innovation in PT programs. However, educators reported that its high-level framing and overlap with existing PT competencies made it difficult to operationalize and distinguish its pain-specific contributions. This domain captures a central tension between the profile’s adaptability, seen as a strength, and its limited prescriptiveness, which left educators uncertain about how to translate broad principles into targeted educational content.

#### Subtheme 1.1: The PEP competency profile is a supportive tool for curriculum innovation

Participants valued the PEP competency profile for its adaptability, simplicity, and rigorous development process, noting that it aligns well with program missions and supports curriculum reflection. Educators described using the profile to justify content inclusion, advocate for resource allocation, and reinforce the importance of pain education.

I wanted to build on what was said about using the competencies as a tool to advocate. […] It helps me justify that we can’t pare things down. (FG 4, session 1, educator 6)

The competency-level framing was seen as a key strength, allowing flexibility in teaching and assessment without imposing prescriptive methods. In this sense, the profile served less as a directive standard and more as a legitimizing reference point, providing institutional weight to pain content that might otherwise be deprioritized. Additionally, the profile’s national endorsement and evidence-based foundation reinforced its credibility as a tool for driving curriculum innovation.

#### Subtheme 1.2: Operationalization is challenging

Though the PEP competency profile is viewed as adaptable, educators reported struggling with its practical application, citing a lack of specificity and detailed guidance. The high-level framing of concepts such as person-centered care and the therapeutic alliance was perceived as too broad for pain-specific applications, making it difficult to develop concrete teaching and assessment strategies.

One challenge I’ve faced with the competency profile is translating it into something practical. I’m developing new content and have the freedom to change topics and formats. The competencies make sense at a high level, but when you get into the practical aspects, […] it leaves a lot to our judgment. (FG 4, session 1, educator 5)

Participants described a need to “fill in the gaps” between the conceptual framing of the profile and day-to-day curricular decisions. This gap between theory and application reflects a broader challenge in HPE, where frameworks must be both general enough to apply across contexts and specific enough to support instructional design. The lack of accompanying tools, such as assessment templates or case-based examples, was viewed as a barrier to meaningful uptake.

#### Subtheme 1.3: Overlap with existing PT competencies

Participants noted significant overlap between the PEP competencies and existing PT curricular elements, particularly in communication, patient-centered care, and the therapeutic alliance.

These are fairly common competencies, just applied to pain. (FG 3, session 1, director 13)

This perceived redundancy had mixed implications. On one hand, it facilitated integration by allowing educators to map existing content onto the PEP framework with minimal disruption. On the other, it raised concerns about the profile’s distinctiveness. Participants questioned whether the PEP competencies added value or simply reframed established skills through a pain-specific lens. For some, this ambiguity risked diminishing the profile’s legitimacy as a curricular innovation. At a deeper level, these concerns point to ongoing tensions in PT education between generalist training and condition-specific expertise. Without clearer articulation of what makes pain management pedagogically unique, the profile’s added value could be diluted, potentially limiting its impact on curriculum development.

### Outer setting domain

This theme captures how regulatory frameworks and external partnerships influence the implementation of the PEP competencies. Participants highlighted a central tension: though accreditation requirements can limit flexibility, they also offer opportunities to legitimize and embed pain competencies. Similarly, external partners were seen as valuable but underleveraged due to institutional and logistical constraints. These external forces shaped what was possible within local curricula, often defining both the windows of opportunity and the practical limits of implementation.

#### Subtheme 2.1: Regulatory demands constrain and enable pain education in PT curricula

Regulatory and accreditation requirements shape PT curricula by setting content priorities, leaving limited space for competencies that are not formally mandated. Pain management was often seen as competing with emerging regulatory priorities such as expanded scope of practice. Despite these constraints, participants identified the alignment between the PEP profile and broader regulatory frameworks, such as the National Physiotherapy Advisory Group (NPAG), as a strategic advantage. This alignment allowed educators to advocate for integration without requiring large-scale curricular reform.

Accreditation standards tie us to the NPAG competency profile, but since the PEP profile aligns well with that, we can effectively implement both at the same time. (FG 3, session 2, director 13)

This suggests that the PEP profile’s uptake may hinge less on its content than on its perceived compatibility with existing institutional mandates. Participants described accreditation and curriculum renewal cycles as strategic entry points for implementation, reinforcing how system-level timing and alignment shape what gets prioritized in practice.

#### Subtheme 2.2: External partners shape PEP competency implementation in PT programs

Patient partners, clinical supervisors, and professional organizations were identified as valuable contributors to pain education, particularly through simulation activities, case studies, and mentorship. However, these partnerships were often described as underused, despite their pedagogical value. Participants cited coordination time, competing priorities (e.g., accreditation demands), lack of compensation, and partner fatigue as barriers to deeper engagement. Faculty also noted that clinical settings and external initiatives, like local Extension for Community Healthcare Outcomes (ECHO) projects, can provide faculty development and interprofessional learning opportunities but often fall outside the formal curriculum.

We have a provincial organization and an ECHO. […] I mention it and give the students resources, but it often falls into the “too much to look at” category. (FG 4, session 2, educator 9)

These comments reflect a structural gap between the perceived value of external input and the systems in place to support it. Without formal mechanisms or incentives for sustained collaboration, these partnerships remain peripheral. Participants emphasized that deeper integration with clinical and community partners will be essential to reinforcing the PEP competencies across learning environments and bridging the gap between classroom and clinical practice.

### Inner setting domain

The third theme highlights how institutional structures and culture, particularly curriculum overload, competing priorities, and faculty dynamics, constrain the cohesive implementation of the PEP competencies. Pain education is often viewed as important but nonurgent, resulting in inconsistent uptake and limited strategic alignment across courses, placements, and faculty roles. These patterns point to a deeper structural issue between the aspirations of curricular innovation and the realities of institutional inertia.

#### Subtheme 3.1: Challenges and strategic opportunities in PT program structures

Participants expressed that program structures play a key role in shaping how pain competencies are implemented, with competency-based curricula aligning more easily with the PEP profile. However, curriculum density and institutional priorities, such as accreditation requirements and equity, diversity, and inclusion (EDI) initiatives, often take precedence.

We’re currently focused on EDI, which keeps the faculty busy. We don’t want to overwhelm them with too many new initiatives. (FG 3, session 2, director 14)

Participants noted that pain education is recognized as important but difficult to prioritize within already saturated curricula. The tendency to add rather than replace content exacerbates this challenge.

We constantly add but rarely remove content. We try to be creative, like promoting self-directed learning to free up time for essential interaction. (FG 3, session 2, director 9)

Participants shared that limited faculty time, stretched budgets, and overlapping academic demands further restrict implementation, leaving little room for additional competencies like pain management. These patterns highlight that, in saturated curricula, perceived importance alone is insufficient to drive adoption. Without clear alignment to institutional priorities, such as accreditation standards or licensure requirements, pain education remains vulnerable to displacement by more visibly strategic initiatives.

#### Subtheme 3.2: Faculty autonomy and institutional culture shape inconsistent implementation of pain competencies

Participants felt that though faculty members generally support pain education, few actively champion its implementation, leaving it largely dependent on individual educators rather than institutional efforts. They described a culture of passive support, where pain competencies are acknowledged as important but are not consistently reinforced across courses, faculty members, or clinical placements.

There’s broad support for pain, but nobody else actively champions it. (FG 1, session 2, educator 13)

Though faculty autonomy was seen as allowing for creativity and responsiveness, it also led to inconsistencies in how pain competencies are addressed. Participants described how traditional technical skills, such as manual therapy, continued to dominate curricular time, limiting the visibility of pain competencies. This reveals a deeper cultural pattern in which innovation relies on individual initiative rather than institutional alignment, making it difficult to sustain consistent messaging or ensure integration across learning environments.

### Individuals domain

This theme highlights how the implementation of PEP competencies relies heavily on a few committed educators, with program directors coordinating resources and priorities, whereas students’ limited exposure to interpersonal skills and the demands of a dense curriculum hinder engagement with pain education. Together, these dynamics reveal the fragility of an implementation model that depends more on individual initiative than on systemic alignment.

#### Subtheme 4.1: PEP implementation relies on a few committed champions

Participants noted that pain education in PT programs relies disproportionately on a handful of faculty members, with some programs lacking strong champions to advocate for it. They emphasized that areas with strong advocates tend to receive more institutional support.

Having a faculty member championing it makes all the difference. Without that, like in my case, it’s harder to give it the attention it needs. (FG 1, session 2, educator 1)

Though local champions can be powerful change agents, participants raised concerns about the sustainability of this model, particularly given the lack of formal institutional support. The burden of implementation often fell on clinicians-turned-educators who felt underprepared to teach complex interpersonal and psychosocial content.

Improving teaching skills is crucial. Many of us come from clinical backgrounds, but teaching is quite different. (FG 4, session 1, educator 14)

These reflections underscore a broader challenge: in the absence of coordinated faculty development or curricular mandates, the success of implementation becomes contingent on individual commitment rather than structural support, making it difficult to scale or sustain change over time.

#### Subtheme 4.2: Program directors coordinate and enable change

Program directors play a pivotal role in implementing pain management competencies by aligning them with institutional goals and curricular priorities. Their responsibilities include coordinating resources, managing faculty workloads, and fostering collaboration among educators.

Leadership has a vital role to play: gathering information, acting as a facilitator to create spaces for people to work together, ensuring consistency, giving them the tools, structuring them, and organizing meetings. (FG 2, session 2, director 4, open translation)

However, even when directors are supportive, their efforts are constrained by systemic factors such as budget limitations, faculty turnover, and competing institutional agendas (e.g., EDI initiatives). These pressures limit continuity and can fragment implementation efforts. Directors who actively support faculty development, facilitate interdisciplinary collaboration, and establish clear communication channels contribute to a more sustainable and cohesive approach to curriculum enhancement.

#### Subtheme 4.3: Student characteristics and curriculum intensity hinder engagement with pain competencies

Student engagement with pain-related competencies is influenced by their preparedness, attitudes, and capacity to integrate interpersonal skills into their training. Participants noted that students often prioritize technical skills over interpersonal competencies, perceiving them as less critical to clinical practice. Educators also highlighted that students often struggle with abstract concepts, such as the therapeutic alliance and patient-centered care.

It’s a challenging subject to teach because of its inherent subjectivity, murkiness, and messiness. Students sometimes feel they have limited bandwidth to engage with that, and it’s quite different from how things are presented in other areas of their training, where it’s more linear. (FG 1, session 2, educator 1)

Educators noted that this resistance was not purely cognitive, but cultural, reflecting the broader values embedded in PT training, where measurable skills are often privileged over relational ones. The intensity of the curriculum further limits space for reflective learning or integration of complex psychosocial content. Together, these factors illustrate how the learning environment can diminish students’ engagement with pain competencies, even when faculty efforts are in place.

### Implementation process domain

This theme captured the notion that the implementation of PEP competencies remains largely conceptual, with implementation strategies focused on curriculum mapping, structured evaluation, and collaboration but lacking systematic application and institutional coordination.

#### Subtheme 5.1: Pain competency implementation demands mapping, strategy, and evaluation

Participants outlined a structured approach typically used to implement new competencies, involving curriculum mapping, gap identification, strategic implementation, and evaluation. This process is seen as critical for ensuring coherence and avoiding redundancy when embedding pain education.

Mapping what’s in existence to the competency profile in the curriculum would be our starting point to see what’s already there. […] Then, the next step would be identifying where the gaps are and […] which course the content would live in. And then, drilling down into where it could live in the learning activities and assessments within that course. (FG 3, session 2, director 1)

Though this approach reflects sound planning, most programs had not yet applied it systematically to the PEP profile. Participants appeared to be in the contemplation or early preparation stages of implementation, constrained by institutional barriers, limited resources, and the absence of structured guidance. This gap between planning and execution underscores the difficulty of translating implementation intent into practice in the absence of formal accountability or systemic support.

#### Subtheme 5.2: The implementation of pain competencies requires a collaborative approach

Collaboration within PT programs is a key facilitator for implementing pain competencies. Open communication, collegial decision making, and shared responsibility help overcome barriers and create opportunities for embedding pain education.

Decisions have to be shared, collaborative decisions. We have to be considerate of other people’s teaching and the impact on students. (FG 1, session 1, educator 5)

In the absence of formal mandates or top-down directives, implementation often relied on internal collaboration to initiate and sustain curricular change. Participants described co-developing teaching tools, such as shared case materials or assessment strategies, as a means of building alignment across instructors and promoting uptake. Some also highlighted the role of students in shaping curriculum design, particularly in refining pedagogical approaches and identifying learning needs.

I think students have a lot to say about the content but especially about the teaching strategies we’re going to use. (FG 5, session 1, educator 6, open translation)

These reflections suggest that collaborative curriculum design, including student input, may be a strategic way to overcome inertia and foster more grounded, context-specific implementation pathways.

### Outcomes addendum

Participants indicated that the PEP competency profile has the potential to transform PT education and patient care, but the lack of rigorous assessment approaches limits the ability to evaluate its impact and ensure its effective implementation. Together, these subthemes highlight a disconnect between the profile’s conceptual influence and the mechanisms needed to embed it meaningfully into educational systems.

#### Subtheme 6.1: The implementation of the PEP competency profile has the potential to transform PT education and patient care broadly

Participants described the anticipated benefits of implementing the PEP competency profile into PT programs. They believed that embedding these competencies could improve curricular alignment, enhance student preparedness to manage complex conditions such as chronic pain, and reinforce the integration of interpersonal competencies like communication and empathy into assessments.

Pain could be a great context for learning certain skills, like narrative medicine or complex condition management that could be applied elsewhere. Understanding pain might help students better understand other complex conditions. (FG 3, session 1, director 14)

Some participants suggested that, in the long term, strengthening pain management education could lead to improved patient-centered care, better patient outcomes, and even greater professional retention.

If more students understood chronic pain and provided effective education to help patients live with persistent pain, we’d see improvements in quality of life. (FG 4, session 1, educator 11)

These reflections underscore the perceived symbolic and strategic value of the PEP profile. However, in the absence of mechanisms to assess or track student learning, this potential remains unrealized.

#### Subtheme 6.2: The lack of rigorous assessment approaches limits the evaluation and uptake of PEP competencies in PT education and practice

Although some participants reported actual efforts to incorporate elements of the PEP competency profile into their teaching, they described its implementation as informal and inconsistent across programs. Most participants noted that competency tracking primarily occurred through curriculum mapping and assessments that categorize exam questions by competency and taxonomic level. However, these measures did not necessarily indicate whether students had developed the intended competencies.

We already have indicators, such as competency mapping in course outlines and exams, with questions categorized by skill and taxonomic level. We know which competencies are being assessed. However, these are not always indicators of success in competency development. (FG 2, session 1, director 4, open translation)

Participants emphasized that the absence of rigorous assessment approaches makes it difficult to evaluate competency acquisition and ensure students are developing the necessary skills. Though clinical placements were identified as key learning opportunities, variation in placement experiences and supervisor expertise made it challenging to assess pain competencies consistently. Some educators reported individual efforts to integrate PEP-related content into their courses, but there was no evidence of coordinated, program-wide adoption. Participants emphasized that rigorous assessment approaches and structured feedback mechanisms are essential next steps in ensuring that pain competencies are not only covered in curricula but also meaningfully assessed in a way that supports student learning across PT programs.

## Discussion

This study highlights the complex nature of the PEP competency profile implementation within Canadian PT programs. It also sheds light on the interplay of institutional, individual, and systemic factors that shape implementation efforts. Though participants recognized the profile’s value in guiding curriculum innovation, implementation efforts were hindered by barriers related to operationalization, limited resources, and structural inertia. These findings align with broader discussions in HPE, where reform is shaped by multiple levels of decision making, institutional constraints, and contextual factors, with evidence alone rarely driving change.^57,71^

The data indicate three interconnected challenges: (1) a lack of structured guidance for teaching and assessing competencies; (2) an overreliance on faculty champions, which highlights the need for systemic institutional support; and (3) the absence of rigorous assessment approaches to evaluate competency acquisition. These challenges are interrelated, because faculty uncertainty about implementation contributes to inconsistent uptake, reliance on individual educators reflects the lack of institutional commitment, and the absence of rigorous evaluation mechanisms complicates efforts to track progress.

Despite broad endorsement of the PEP profile, its implementation remains a challenge. Participants acknowledged that though the competencies align with existing curricula, their high-level framing and overlap with other professional competencies (e.g., patient-centered care) made it difficult to translate into concrete teaching and assessment strategies. This challenge may stem, in part, from the intentionally broad scope of the PEP competencies, which emphasize overarching principles rather than detailed instructional guidance, with the expectation that future work would provide more practical direction.^48^ This reflects a common challenge in curricular reform: the need to balance adaptability with sufficient specificity to ensure effective uptake.^72–77^ Though adaptability enables institutions to tailor implementation to their contexts, the absence of clear, structured guidance creates uncertainty and can hinder adoption.^72–77^ These challenges are compounded by structural factors, such as the underrecognition of relational competencies in clinical training and the long-standing disciplinary hierarchies that can marginalize interpersonal and person-centered competencies. To support implementation, shared resources (e.g., teaching strategies, assessment templates, and instructional content) could be developed collaboratively at a national level. External partners, professional organizations, and inter-institutional networks could further enhance capacity by facilitating partnerships with people living with pain and developing extracurricular student learning opportunities. National collaboration could also be strengthened through initiatives such as a centralized online repository of educational resources and formal communities of practice that foster shared problem solving, mentoring, and pedagogical innovation across PT programs.

Another major challenge was the overreliance on faculty champions to lead implementation. Though engaged educators played a critical role in advocating for pain education, their efforts were often informal and lacked integration into structured institutional strategy. Without dedicated resources or explicit inclusion into accreditation requirements, the sustainability of these efforts remains uncertain. Similar patterns have been noted in the literature, where lasting implementation requires broad faculty engagement and administrative buy-in rather than isolated efforts.^78–80^ Although program directors are central to coordinating curriculum change, participants reported that competing priorities (e.g., accreditation demands or EDI initiatives) often overshadowed the integration of new competencies. These constraints, including faculty workload and institutional inertia, are well-documented barriers in HPE.^81–84^ To move beyond faculty-driven efforts, pain competencies could be embedded within accreditation frameworks, national curriculum standards, and reforms to entry-to-practice licensure. Structured faculty development, institutional capacity-building strategies, and support from professional organizations could further facilitate implementation.^85–87^ In the short term, this may include workshops on instructional strategies, peer mentoring, and collaborative teaching tools such as shared case libraries or simulation scenarios. These strategies could improve consistency and reduce reliance on individual champions. Rather than treating pain management as an add-on, integrating into existing curricular structures may ease faculty burden and promote uptake. A phased model, starting with curriculum mapping, faculty leads, or cross-departmental working groups, could support early momentum. However, long-term sustainability will require alignment with accreditation standards, national reforms, and shared evaluation tools, shifting pain education from isolated efforts to embedded, system-level adoption.

An important gap identified in this study was the lack of rigorous assessment approaches to measure student competency in pain management, a concern echoed in the literature.^42,88^ Though some programs had begun competency mapping, these efforts do not necessarily assess whether students are developing the intended competencies.^22,89,90^ Without structured evaluation approaches, it remains unclear how effectively PEP competencies translate into clinical preparedness. Competency development in complex areas, such as pain management, occurs over time, is dynamic, and benefits from a combination of assessment methods that reflect real-world application.^22,89–94^ Though traditional formats such as multiple-choice examinations can assess foundational knowledge, they may be insufficient on their own to capture the depth of clinical reasoning and patient-centered approaches required in practice.^95–98^ A more meaningful approach would combine simulation-based assessments, structured clinical observation, and case-based evaluations across the educational trajectory.^99–102^ Establishing clear milestones specific to the PEP competencies would further support a progressive and structured evaluation of students’ abilities to apply pain management principles in clinical settings, ensuring entry-level competence.^103–105^

The CFIR provided valuable insights into the multilevel barriers and facilitators shaping competency implementation in PT education. However, curriculum change differs from the discrete interventions for which CFIR was originally designed, because it is inherently iterative, decentralized, and shaped by intersecting institutional and faculty-driven influences.^57,71^ Curriculum change often evolves through cycles of adaptation, faculty discretion, and institutional negotiations rather than following a linear or standardized process.^71,106,107^ CFIR’s emphasis on structured stages and planned processes does not fully capture this reality, and its outcome constructs assume a level of standardization that is often impractical in educational contexts.^57,71,107^ These challenges align with broader critiques in HPE, where scholars have called for frameworks that better account for the dynamic, nonlinear nature of pedagogical change.^71,106^ Future research should consider adapting the CFIR, by expanding its constructs to better capture iterative curriculum refinement and informal decision making. More broadly, we call on implementation science researchers to join forces with HPE scholars to develop models and frameworks that are more suitable for these contexts.

Though these findings highlight key challenges and opportunities in implementing the PEP competency profile, several limitations should be considered. This study relied on self-reported perceptions from faculty and program directors, which may not fully capture the experiences of students or the practical challenges they face in developing pain management competencies. Additionally, this study did not explore the impact of informal learning experiences or the hidden curriculum, which likely shape how students acquire and apply these competencies.^108–110^ Future research should include student perspectives to better understand how pain competencies are internalized, enacted in clinical contexts, and shaped by both formal and informal socialization. Although attributing patient outcomes to educational interventions is methodologically complex, longitudinal studies could explore whether structured implementation of the PEP profile enhances competency development and improves clinical outcomes. Such research would help clarify the downstream impact of competency adoption and inform best practices for strengthening pain education in PT programs.

## Conclusion

This study highlights key challenges and opportunities in implementing the PEP competency profile within Canadian PT programs. Though the profile offers a structured framework for strengthening pain management education, its successful implementation remains influenced by institutional structures, faculty engagement, and the availability of clear implementation and assessment strategies. The findings underscore the need for structured guidance to support educators in translating competencies into curriculum, sustainable institutional support beyond individual faculty champions, and rigorous assessment approaches to ensure competency acquisition. Addressing these barriers will be critical for fostering a more consistent and comprehensive approach to pain education across PT programs. Beyond identifying implementation challenges, this study contributes to a broader understanding of how competency frameworks are adopted within HPE. The application of CFIR provided valuable insights into multilevel factors shaping curriculum change but also highlighted the need to refine implementation models to better capture the iterative nature of educational integration. By sharing potential strategies to support implementation, this work lays the foundation for improving pain education in PT programs, ultimately strengthening the ability of graduates to provide high-quality, patient-centered pain management.

## Supplementary Material

Supplementary Material 1.pdf

Supplementary Material 2_pdf version.docx
