# Supplementary material for: Implementing pain competencies in Canadian physiotherapy education: Challenges, barriers, and opportunities
Source: Can J Pain. 2025 Dec 15;9(1):2574969. doi: 10.1080/24740527.2025.2574969 (PMC12707519; doi:10.1080/24740527.2025.2574969)
Supplement: Supplementary Material 2_pdf version.docx [file UCJP_A_2574969_SM2782.docx]

| **CFIR Domains** | **CFIR constructs** | **Quotes** | **Subtheme name** | **Subtheme description** |
| --- | --- | --- | --- | --- |
| **Innovation:** The first theme highlights that the PEP competency profile was viewed by participants as a flexible and structured tool that could support curriculum innovation in PT programs. However, educators reported that its high-level framing and overlap with existing PT competencies made it difficult to operationalize and distinguish its pain-specific contributions. | Innovation Design Innovation Source Innovation Evidence-Base Innovation Relative Advantage Innovation Adaptability | The presence of interpersonal competencies as a standalone feature of the profile has given permission or allowed me to focus on integrating these more specifically. (FG 1, Session 1, Educator 1) The idea that it’s stated at a competency level and not at a very granular skills, knowledge, attitudes, or values level, allows a lot of flexibility or adaptability because it’s just setting a target, but not really telling you how to get there. (FG 1, Session 1, Educator 14) Je pense que c’est relativement rare qu’il y ait eu un processus aussi formel et rigoureux pour bonifier un contenu de formation dans un programme de physiothérapie canadienne. […] Globalement, ça donne beaucoup de crédibilité et un certain poids aux propositions qui sont faites. (FG 5, Session 1, Educator 8) I wanted to build on what was said about using the competencies as a tool to advocate. […] It helps me justify that we can’t pare things down. (FG 4, Session 1, Educator 6) | Subtheme 1.1: The PEP competency profile is a supportive tool for curriculum innovation. | Participants valued the PEP competency profile for its adaptability, simplicity, and rigorous development process, noting that it aligns well with program missions and supports curriculum reflection. Educators described using the profile to justify content inclusion, advocate for resource allocation, and reinforce the importance of pain education. The competency-level framing was seen as a key strength, allowing flexibility in teaching and assessment without imposing prescriptive methods. Additionally, the profile’s national endorsement and evidence-based foundation reinforced its credibility as a tool for driving curriculum innovation. |
|  | Innovation Complexity Innovation Trialability Innovation Design | Ce serait intéressant de proposer des outils spécifiques, des jalons de compétence et des cadres d’évaluation. Nos enseignants ont besoin d’outils pédagogiques concrets pour aller plus loin dans leur réflexion et fournir des balises aux étudiants. (FG 2, Session 1, Director 4) One challenge I’ve faced with the competency profile is translating it into something practical. I’m developing new content and have the freedom to change topics and formats. The competencies make sense at a high level, but when you get into the practical aspects, […] it leaves a lot to our judgment. (FG 4, Session 1, Educator 5) Ce qui serait vraiment utile, c’est de contextualiser les compétences spécifiques à la douleur. Par exemple, pour une compétence comme "faciliter les transitions de soins", il faudrait détailler ce que cela signifie dans le contexte d’une personne avec douleur chronique. (FG 2, Session 1, Director 5) | Subtheme 1.2: Operationalization is challenging. | While the PEP competency profile is viewed as adaptable, educators reported struggling with its practical application, citing a lack of specificity and detailed guidance. The high-level framing of concepts such as person-centered care and the therapeutic alliance was perceived as too broad for pain-specific applications, making it difficult to develop concrete teaching and assessment strategies. Educators found it challenging to balance theoretical depth with practical implementation. Participants emphasized the need for clearer guidance, structured resources, and practical tools to bridge the gap between the profile’s theoretical framework and the realities of educational practice. |
|  | Innovation Relative Advantage Innovation Adaptability | De mon point de vue pédagogique, il est important de s’assurer que ces compétences transversales soient bien mises en œuvre dans des familles de situations emblématiques de problèmes de douleur. Par exemple, est-ce qu’il y a des éléments spécifiques de l’alliance thérapeutique à adresser lorsqu’on parle de la gestion de la douleur, par rapport à d’autres situations? (FG 2, Session 2, Director 5) These are fairly common competencies, just applied to pain. (FG 3, Session 1, Director 13) | Subtheme 1.3: Overlap with existing PT competencies. | Participants noted significant overlap between the PEP competencies and existing PT curricular elements, particularly in communication, patient-centered care, and the therapeutic alliance. While this alignment facilitated implementation, it also raised concerns about whether the profile provided distinct value. The broad applicability of the profile was seen as both a facilitator and a challenge, as it allowed for easier incorporation into existing curricula but also risked redundancy. Participants emphasized the importance of clearly defining the competencies that are unique to pain management to reinforce the profile’s distinct contribution. Participants added that, without clear differentiation, the profile’s added value could be diluted, potentially limiting its impact on curriculum development. |
| **Outer setting:** The theme in the outer setting captured the influence of regulatory standards and external partnerships on the implementation of the PEP competencies. Accreditation requirements shaped institutional priorities, while external partnerships were seen as underutilized opportunities to enhance pain education. | Policies & Laws External Pressure | La première préoccupation reste de bien répondre aux organismes réglementaires et aux nouvelles pratiques. Nous devons nous mobiliser pour que les étudiants soient compétents sur ces aspects. (FG 2, Session 2, Director 3) In our province, we’re dealing with scope of practice changes that need to be implemented in the curriculum. Those will take priority for now. (FG 3, Session 2, Director 14) Accreditation standards tie us to the NPAG competency profile, but since the PEP profile aligns well with that, we can effectively implement both at the same time. (FG 3, Session 2, Director 13) | Subtheme 2.1: Regulatory demands constrain and enable pain education in PT curricula. | Regulatory and accreditation requirements shape PT curricula by setting content priorities, limiting flexibility for additional competencies such as pain management. Despite these constraints, the alignment between the PEP profile and broader regulatory frameworks, such as the NPAG, was seen as a potential facilitator, allowing for implementation without major curricular restructuring. Participants noted that scope of practice changes (e.g., X-ray prescription) and evolving professional standards often take precedence, making it difficult to introduce new competencies unless they directly align with accreditation mandates. Curriculum renewal and accreditation cycles were identified as key opportunities to implement pain education without disrupting existing structures. |
|  | Partnerships & Connections Local Attitudes Societal Pressure Local Conditions | I can teach it, and we can integrate it into case studies, but do they get to practice it? Do they get to follow someone for weeks and really integrate and understand it? I’m not sure all our students necessarily get those opportunities in placement. (FG 4, Session 2, Educator 8) We have a provincial organization and an ECHO. […] I mention it and give the students resources, but it often falls into the "too much to look at" category. (FG 4, Session 2, Educator 9) I definitely notice a difference when we’re working on an interpersonal activity and there’s a patient involved. […] When students hear a patient’s story or plan an assessment or treatment based on what a patient says, and then get feedback from the patient, engagement is really high. They seem to understand the importance of validation. (FG 1, Session 2, Educator 1) | Subtheme 2.2: External partners shape PEP competency implementation in PT programs. | Patient partners, clinical supervisors, and professional organizations provide valuable opportunities to reinforce pain competencies through simulation activities, case studies, and mentorship. However, logistical barriers (e.g., the financial cost of compensating participants or the time required to coordinate partnerships) and competing priorities of various partners often limit engagement and reduce the impact of these collaborations. Similarly, while initiatives like local Extension for Community Healthcare Outcomes (ECHO) projects provide faculty development and interprofessional learning opportunities, these resources remain underutilized. Participants emphasized the need for sustained partnerships and structured collaboration to bridge gaps between academic and clinical education and ensure students are ready to meet the complexities of pain management. |
| **Inner setting**: The third theme highlighted how competing institutional priorities and passive faculty support create significant barriers to implement PEP competencies, with inconsistent messaging across courses and limited curriculum space restricting cohesive implementation. | Compatibility Relative Priority Tension for Change Available Resources (Funding, Space, Materials & Equipment)  Structural characteristics (Work Infrastructure) Mission Alignment Access to Knowledge & Information | The second domain of the competency profile (communication, patient-centered care, etc.) is easier to integrate since it overlaps with existing curriculum. (FG 3, Session 2, Director 13) We’re currently focused on EDI, which keeps the faculty busy. We don’t want to overwhelm them with too many new initiatives. (FG 3, Session 2, Director 14) We constantly add but rarely remove content. We try to be creative, like promoting self-directed learning to free up time for essential interaction. (FG 3, Session 2, Director 9) I don’t think I’ve ever been explicitly told, “No, you can’t do this because we don’t have enough money.” But budget is probably the first item on the agenda at all our meetings. There’s an understanding that things are tight. (FG 1, Session 2, Educator 2) | Subtheme 3.1: Challenges and strategic opportunities in PT program structures. | Participants expressed that program structures play a key role in shaping how pain competencies are implemented, with competency-based curricula aligning more easily with the PEP profile. However, curriculum density and institutional priorities, such as accreditation requirements and equity, diversity, and inclusion (EDI) initiatives, often take precedence. Participants noted that pain education is recognized as important but difficult to prioritize within already saturated curricula. The tendency to add rather than replace content exacerbates this challenge. Participants shared that limited faculty time, stretched budgets, and overlapping academic demands further restrict implementation, leaving little room for additional competencies like pain management. |
|  | Relational Connections Culture (Deliverer-Centeredness, Recipient-Centeredness, Learning-Centeredness) Communications | I think there was generally an appreciation of the importance of pain in curricula. […] I also feel that there’s a culture of “this is important, but I don’t really want to touch this.” (FG 1, Session 1, Educator 12) In my program’s culture, there’s a broadly “live and let live” attitude. There’s broad support for pain, but nobody else actively champions it. (FG 1, Session 2, Educator 13) There’s a lack of interest and emphasis on interpersonal skills. In my pain course, based on students’ reactions, I think we’re doing well in addressing those competencies, but historically, there hasn’t been much emphasis. (FG 1, Session 2, Educator 1) | Subtheme 3.2: Faculty autonomy and institutional culture shape inconsistent implementation of pain competencies. | Participants felt that, while faculty members generally support pain education, few actively champion its implementation, leaving it largely dependent on individual educators rather than institutional efforts. They described a culture of passive support, where pain competencies are acknowledged as important but are not consistently reinforced across courses, faculty members, or clinical placements. Additionally, faculty autonomy allows for innovation but also leads to inconsistencies in how pain competencies are addressed. Traditional technical skills, such as manual therapy, often take precedence in program structures, reinforcing a fragmented approach to pain education. |
| **Individuals**: This theme highlights how the implementation of PEP competencies relies heavily on a few committed educators, with program directors coordinating resources and priorities, while students’ limited exposure to interpersonal skills and the demands of a dense curriculum hinder engagement with pain education. | Characteristics (Need, Capability, Opportunity, Motivation) Roles (Implementation Leads, Implementation Facilitators, Innovation Deliverers, Other Implementation Support) | Having a faculty member championing it makes all the difference. Without that, like in my case, it’s harder to give it the attention it needs. (FG 1, Session 2, Educator 1) Improving teaching skills is crucial. Many of us come from clinical backgrounds, but teaching is quite different. […] I think the biggest barrier might be myself, as I try to improve how I teach these competencies. (FG 4, Session 1, Educator 14) Je pense qu’il y a une complexité à enseigner cela. C’est un profil avec beaucoup de compétences transversales qui s’acquièrent avec l’expérience. Comment enseigner cela peut être très difficile, notamment au niveau de la communication avec les patients et les notions de justice sociale. (FG 5, Session 1, Educator 9) | Subtheme 4.1: PEP implementation relies on a few committed champions. | Participants noted that pain education in PT programs relies disproportionately on a handful of faculty members, with some programs lacking strong champions to advocate for it. They emphasized that areas with strong advocates tend to receive more institutional support. However, this over-reliance on a few individuals presents sustainability concerns. Additionally, many educators come from clinical backgrounds and feel ill-equipped to teach complex competencies, such as communication, social justice, and patient-centered care. |
|  | Roles (High-level Leaders, Other Implementation Support) | La direction a un rôle primordial: bien recenser l’information, agir comme facilitateur pour donner des espaces pour que les gens collaborent, que ce soit cohérent, leur donner les outils, les structurer, organiser des réunions. (FG 2, Session 2, Director 4) If my program director were here, I would also say that our leadership is very supportive. (FG 1, Session 2, Educator 1) | Subtheme 4.2: Program directors coordinate and enable change. | Program directors play a pivotal role in implementing pain management competencies by aligning them with institutional goals and curricular priorities. Their responsibilities include coordinating resources, managing faculty workloads, and fostering collaboration among educators. However, competing priorities, resource limitations, and faculty turnover create challenges that can disrupt continuity. Directors who actively support faculty development, facilitate interdisciplinary collaboration, and establish clear communication channels contribute to a more sustainable and cohesive approach to curriculum enhancement. |
|  | Roles (Innovation Recipients) | It’s a challenging subject to teach because of its inherent subjectivity, murkiness, and messiness. Students sometimes feel they have limited bandwidth to engage with that, and it’s quite different from how things are presented in other areas of their training, where it’s more linear. (FG 1, Session 2, Educator 1) Mais on sent encore que beaucoup de ceux qui s’inscrivent en physiothérapie ont une approche très biomécanique et anatomique. Acquérir ces autres compétences reste un défi. (FG 2, Session 1, Director 3) | Subtheme 4.3: Student characteristics and curriculum intensity hinder engagement with pain competencies. | Student engagement with pain-related competencies is influenced by their preparedness, attitudes, and capacity to integrate interpersonal skills into their training. Participants noted that students often prioritize technical skills over interpersonal competencies, perceiving them as less critical to clinical practice. Educators also highlighted that students often struggle with abstract concepts, such as the therapeutic alliance and patient-centered care. Additionally, the intensity of PT curricula leaves students overwhelmed, limiting their ability to fully engage with non-technical competencies. |
| **Implementation process**: This theme captured the notion that the implementation of PEP competencies remains largely conceptual, with implementation strategies focused on curriculum mapping, structured evaluation, and collaboration, but lacking systematic application and institutional coordination. | Planning Reflecting and Evaluating (Innovation, Implementation) Assessing Context Assessing Needs (Innovation Deliverers) | La première étape, c’est de s’assurer qu’on a un alignement avec le référentiel, puis nos objectifs d’apprentissage. […] Ensuite, c’est de regarder les manques à accomplir et planifier ces manques pour s’assurer qu’on a une cohérence au niveau d’une approche programme. […] Finalement, c’est de penser aux indicateurs de réussite du projet. S’assurer que les retombées soient alignées avec nos objectifs initiaux. (FG 2, Session 2, Director 3) Mapping what’s in existence to the competency profile in the curriculum would be our starting point to see what’s already there. [...] This would have to be at the level of program objectives, course objectives, teaching and learning activities and assessments to see what is included.[...] Then, the next step would be identifying where the gaps are and [...] which course the content would live in. And then, drilling down into where it could live in the learning activities and assessments within that course. (FG 3, Session 2, Director 1) | Subtheme 5.1: Pain competency implementation demands mapping, strategy, and evaluation. | Participants outlined a structured approach typically used to implement new competencies, involving curriculum mapping, gap identification, strategic implementation, and evaluation. This process is seen as critical for ensuring coherence and avoiding redundancy when embedding pain education. However, discussions around these steps remained largely theoretical, as most programs had not yet systematically applied them to the PEP competency profile. |
|  | Assessing Needs (Innovation Recipients) Adapting Doing Teaming Engaging (Innovation Recipients, Innovation Deliverers) Tailoring Strategies | Je pense que les étudiants ont leur mot à dire. Notamment, je trouve que les étudiants ont beaucoup à dire sur le contenu, mais surtout sur les stratégies pédagogiques qu’on va mobiliser. (FG 5, Session 1, Educator 6) Decisions have to be shared, collaborative decisions. We have to be considerate of other people’s teaching and the impact on students. (FG 1, Session 1, Educator 5) C’est sûr que dans mon cas, il faudrait vraiment que je mobilise mes collègues et en faire une affaire de groupe plutôt que quelque chose de personnel. (FG 5, Session 1, Educator 8) | Subtheme 5.2: The implementation of pain competencies requires a collaborative approach. | Collaboration within PT programs is a key facilitator for implementing pain competencies. Open communication, collegial decision-making, and shared responsibility help overcome barriers and create opportunities for embedding pain education. The co-creation of shared pedagogical resources across programs was identified as a promising strategy to streamline implementation. Participants also emphasized that students play an active role in shaping pain education, particularly in refining teaching strategies to enhance engagement. |
| **Outcomes**: Participants indicated that the PEP competency profile has the potential to transform PT education and patient care, but the lack of rigorous assessment approaches limits the ability to evaluate its impact and ensure its effective implementation. | Anticipated Implementation Outcomes  Actual Implementation Outcomes | Exactement, cela renforcerait une culture où les patients sont vus dans leur globalité […]. L’alliance thérapeutique, par exemple, est une compétence transversale qui […] aurait un impact positif non seulement sur la gestion de la douleur, mais aussi sur d’autres aspects des soins. (FG 2, Session 1, Director 5) Pain could be a great context for learning certain skills, like narrative medicine or complex condition management that could be applied elsewhere. Understanding pain might help students better understand other complex conditions. (FG 3, Session 1, Director 14) I believe it would improve patient experiences with healthcare providers. Patients would feel validated, not dismissed, and there would be better therapeutic alliances. If more students understood chronic pain and provided effective education to help patients live with persistent pain, we’d see improvements in quality of life. (FG 4, Session 1, Educator 11) | Subtheme 6.1: The implementation of the PEP competency profile has the potential to transform PT education and patient care broadly. | Participants described the anticipated benefits of implementing the PEP competency profile into PT programs. They believed that embedding these competencies could improve curricular alignment, enhance student preparedness to manage complex conditions such as chronic pain, and reinforce the integration of interpersonal competencies like communication and empathy into assessments. Some participants suggested that, in the long term, strengthening pain management education could lead to improved patient-centered care, better patient outcomes, and even greater professional retention. However, they acknowledged that these impacts remain hypothetical, as the competency profile has yet to be systematically implemented into most programs. |
|  | Actual Implementation Outcomes (Adoption) Innovation Outcomes (Deliverer Impacts, Recipient Impacts) | Since our pain curriculum is spread throughout the two years, having a final evaluation allowed us to see if students could synthesize their knowledge and apply it in clinical scenarios. This evaluation was beneficial for both the students and instructors, providing insights into their integration of knowledge. (FG 4, Session 1, Educator 6) Par exemple, on pourrait vérifier si les étudiants se sentent compétents en stage avec des cas de douleur chronique. (FG 2, Session 1, Director 5) Nous avons déjà des indicateurs, comme le mapping des compétences dans les plans de cours et les examens, avec des questions catégorisées par compétence et niveau taxonomique. Nous savons quelles compétences sont évaluées. Cependant, ce ne sont pas toujours des indicateurs de succès du développement de la compétence. (FG 2, Session 1, Director 4) La partie de validation au niveau de l’implantation inclue le fait de s’assurer avec notre comité d’évaluation si les étudiants performent bien, si les objectifs sont atteints, si les objectifs sont clairement définis et bien intégrés au sein du parcours. (FG 5, Session 2, Educator 7) | Subtheme 6.2: The lack of rigorous assessment approaches limits the evaluation and uptake of PEP competencies in PT education and practice. | Although some participants reported actual efforts to incorporate elements of the PEP competency profile into their teaching, they described its implementation as informal and inconsistent across programs. Most participants noted that competency tracking primarily occurred through curriculum mapping and assessments that categorize exam questions by competency and taxonomic level. However, these measures did not necessarily indicate whether students had developed the intended competencies. Participants emphasized that the absence of rigorous assessment approaches makes it difficult to evaluate competency acquisition and ensure students are developing the necessary skills. While clinical placements were identified as key learning opportunities, variation in placement experiences and supervisor expertise made it challenging to assess pain competencies consistently. Some educators reported individual efforts to integrate PEP-related content into their courses, but there was no evidence of coordinated, program-wide adoption. Participants emphasized that rigorous assessment approaches and structured feedback mechanisms are essential next steps in ensuring that pain competencies are not only covered in curricula but also meaningfully assessed in a way that supports student learning across PT programs. |
